# Expression of lncRNAs *NEAT1* and *lnc-DC* in Serum From Patients With Behçet’s Disease Can Be Used as Predictors of Disease

**DOI:** 10.3389/fmolb.2021.797689

**Published:** 2022-01-19

**Authors:** Shereen Rashad Mohammed, Omayma O. Abdelaleem, Fatma A. Ahmed, Ahmed Ali Abdelaziz, Hoda Abdelbadie Hussein, Hanaa M. Eid, Marwa Kamal, Mostafa Ahmed Ezzat, Marwa A. Ali

**Affiliations:** ^1^ Department of Medical Biochemistry and Molecular Biology, Faculty of Medicine, Fayoum University, Fayoum, Egypt; ^2^ Department of Medical Microbiology and Immunology, Faculty of Medicine, Fayoum University, Fayoum, Egypt; ^3^ Departments of Rheumatology and Rehabilitation, Faculty of Medicine, Al-Azhar University, Cairo, Egypt; ^4^ Department of Internal Medicine, Faculty of Medicine, Fayoum University, Fayoum, Egypt; ^5^ Department of Clinical Pharmacy, Faculty of Pharmacy, Fayoum University, Fayoum, Egypt; ^6^ Department of Clinical Pathology, Faculty of Medicine, Fayoum University, Fayoum, Egypt

**Keywords:** Behcet’s disease, lnc-DC, NEAT1, RT-PCR, lncRNAs

## Abstract

**Background:** Behçet’s disease (BD) is a chronic autoimmune disease. The early diagnosis of BD is very important to avoid serious and/or fatal complications such as eye damage, severe neurological involvement, and large vessel occlusion. New, sensitive biomarkers would aid in rapid diagnosis, the monitoring of disease activity, and the response to treatment.

**Methods:** This study’s aim is to identify two immune system-related BD biomarkers. We measured long non-coding RNAs (lncRNAs) *NEAT1* (nuclear-enriched abundant transcript 1), and *lnc-DC* (lncRNA in dendritic cells) in serum by real-time polymerase chain reaction (RT-PCR) in 52 BD patients and 52 controls. We analyzed the association between *NEAT1* and *lnc-DC* and the clinical parameters of BD. Receiver operating characteristic (ROC) curve analysis was performed to explore the diagnostic performance of the studied genes.

**Results:** Compared to controls, the significant upregulation of *NEAT1* {median [interquartile range (IQR)] = 1.68 (0.38–7.7), *p <* 0.0001} and downregulation of *lnc-DC* [median (IQR) = 0.2 (0.12–1.39), *p =* 0.03] were detected in the sera collected from BD patients. Higher serum expression levels of *NEAT1* and *lnc-DC* were significantly associated with the following clinical presentations: cutaneous lesions, vascular manifestations, articular manifestations, neurological manifestations, and higher disease activity score. Also, high *NEAT1* levels were significantly associated with a negative pathergy test, while higher *lnc-DC* was significantly associated with a positive family history. ROC curves showed that *NEAT1* and *lnc-DC* levels in serum could be used as predictors of BD with high specificity and fair sensitivity. *NEAT1* had an area under the curve (AUC) of 0.692 (95% CI: 0.591–0.794, *p* = 0.001), and *lnc-DC* had an AUC of 0.615 (95% CI: 0.508–0.723, *p* = 0.043).

**Conclusion:** Serum lncRNAs *NEAT1* and *lnc-DC* are biomarkers for BD.

## Introduction

Behçet’s disease (BD) is a chronic multi-systemic inflammatory disease with a wide spectrum of clinical presentations varying from mucocutaneous ulcerations and ocular inflammation to more severe and potentially fatal manifestations such as the vascular system, nervous system, and gastrointestinal tract lesions. Different theories posit that BD is a result of immune system dysfunction due to environmental or infectious triggering factors in genetically susceptible individuals. To date, there is no specific laboratory test for BD diagnosis, and so the identification of essential genes underlying the susceptibility, activity status, or manifestations of the disease is of great importance and may provide new regimens for controlling or treating the disease (Leccese and Alpsoy 2019).

Long non-coding RNAs (lncRNAs) are a group of RNAs that are longer than 200 nucleotides in length and do not encode proteins (Wei et al., 2017). Although the function of most lncRNAs is unclear, numerous studies have documented pivotal functions for lncRNAs in human immune diseases through the activation, differentiation, and imbalanced expression of immune cells (T cells, B cells, macrophages, and natural killer cells) that are implicated in the pathogenesis of various autoimmune diseases such as psoriasis, rheumatoid arthritis (RA), and systemic lupus erythematosus (SLE) (Sigdel et al., 2015). Among these biomarkers are lncRNA *NEAT1* (nuclear-enriched abundant transcript 1) (Imamura et al., 2014; Zhang et al., 2016; Huang et al., 2017; Pandey et al., 2017; Huang et al., 2018; Prinz et al., 2019; Zhang et al., 2019), and *lnc-DC* (lncRNA in dendritic cells) (Wu et al., 2017)].

*NEAT1* is an lncRNA that localizes to and plays a critical role in maintaining the formation of paraspeckles, an interchromatin nuclear body with unknown function (Zhang et al., 2019). Various stimuli that activate inflammasomes can also trigger the expression of *NEAT1*, such as viral infections and tumor suppressor p53 (Adriaens et al., 2016; Zhang et al., 2019). Inflammasome-inducing signals stimulate the release of *NEAT1* from paraspeckles and their translocation to the cytoplasm, where they participate in inflammasome activity. *NEAT1* stimulates one or more innate pattern recognition receptors (PRRs) (Zhang et al., 2019). In addition, *NEAT1* is crucial for inflammasomes and caspase-1 activation, cytokine production, and pyroptotic cell death. *NEAT1* also regulates the expression of a group of chemokines and cytokines, including IL-6 and CXCL10, through the MAPK pathway (Sigdel et al., 2015; Zhang et al., 2019), suggesting a role in the pathogenesis of BD.

*Lnc-DC* is expressed in dendritic cells (DCs) and mediates DC maturation by phosphorylating signal transducer and activator of transcription 3 (STAT3) (Zhang et al., 2020b). As such, *lnc-DC* can induce the differentiation and maturation of DCs, the transcription of immune response genes, and the production of related cytokines, such as TNF-a, IFN-c, IL-13, IL-5, IL-4, and IL-12 (Wang et al., 2014).

Based on this background, we investigated the relationship between the expression levels of two immunity-related lncRNAs, *NEAT1* and *lnc-DC*, with BD susceptibility, activity, and clinical manifestations. We also determined the diagnostic performance of *NEAT1* and *lnc-DC* in differentiating BD patients from healthy controls.

## Subject and Methods

### Patients and Controls

This study was conducted per the Declaration of Helsinki and was approved by the Ethical Committee of the Faculty of Medicine, Fayoum University (protocol code M400). Written consent was obtained from all participants after the declaration of all involved procedures and steps. Participants were categorized into two groups. For Group I, we selected 40 male and 12 female patients fulfilling the criteria of the International Study Group (ISG) for the diagnosis of BD (INTERNATIONAL STUDY GROUP FOR BEHC 1990). Participants were recruited from outpatients’ clinics, patients admitted to the Rheumatology and Rehabilitation Department and/or the Internal Medicine Department of Fayoum University Hospital (Fayoum, Egypt) from June 2019 to January 2021. Patients with other concurrent diseases such as hepatic, renal dysfunction, autoimmune diseases, atherosclerosis, coronary artery disease, essential hypertension, and diabetes were excluded. For Group II, we included 52 age- and sex-matched healthy controls without acute or chronic disease in the study.

Medical history and clinical examination were performed for all patients to detect disease features and complications. Patients underwent a complete medical history (age, sex, duration of the disease, family history), general examination, specific examination to detect the symptoms and signs of BD disease, involving oral or genital ulcers, the presence of cutaneous lesions, Duplex and color-coded Doppler examination on arterial and venous systems, and fundus examination by slit lamp. Central nervous system (CNS) and joint examination by computed tomography and magnetic resonance imaging were performed when indicated to detect any vascular, ocular, CNS, or articular lesions. The pathergy test was done for all patients, and a positive pathergy test was indicated by the formation of a small papule or pustule (3–10 mm) within 1 or 2 days at the site of sterile needle prick (El Boghdady and Shaker 2019). All patients received steroids and colchicine, while 92.3% received azathioprine. Patients were assessed for disease activity using the Behçet’s disease Current Activity Index (BDCAI) score, which is calculated based on the answers to 12 questions regarding disease manifestations over the previous 4 weeks. Active disease scores range from 0 to 12. A higher score indicates a higher level of disease activity (Bhakta et al., 1999). Accordingly, patients were divided into active (patients who met at least one criterion of the ISG for BD) or inactive (no lesions for the previous 4 weeks or more) patients at the time of the study. In the current study, 36 patients (69.3%) were in active disease, of which 38.5% had an activity score of 1, 23.1% had an activity score of 2, and 7.7% had an activity score of 3.

### Sample Collection, RNA Extraction, cDNA Synthesis, RT-PCR Performance, and Calculation of Target Genes’ Fold Changes

Venous blood samples were obtained from all patients and healthy individuals under sterile conditions. The collected venous blood was kept undisturbed at room temperature to allow clotting. The samples were subsequently subjected to centrifugation for serum separation. The clear supernatant present in the upper part of the tube (serum) was transferred to a clean tube and stored in the freezer at −80°C until RNA extraction.

Total RNA, including *NEAT1* and *lnc-DC*, was extracted from the serum using the miRNeasy extraction kit (Qiagen, Valencia, CA, United States) by following the manufacturer’s protocol.

The steps of RNA extraction include the following: first, we cleaned the working place with 70% ethanol. Then, we added 1 ml QIAzol lysis reagent to a 200 μl sample and incubated it for 5 min at room temperature. Next, we added 200 μl chloroform, vortexed the tube for 15 s, and incubated the mixture at room temperature for 2 min. Afterwards, we used cool centrifugation to avoid the destruction of RNA at 120,00× *g* for 15 min at 4°C. Next, we transported the upper aqueous phase to a new collection tube, followed by the addition of 1.5 times its volume of 100% ethanol. After that, we pipetted 750 μl of the mix to the 2 ml RNeasy Mini spin column and centrifuged it at 8,000 ×*g* at room temperature for 15 s. When the mixture had totally passed the column, we discarded the flow through water and reused the tube to repeat this step with the remaining part of the mix. Then, we used the washing buffers in the kit (RWT and RPF) as follows: firstly, we added 700 μl of the RWT buffer to each spin column. Then, we centrifuged it at 8,000 ×*g* at room temperature for 15 s, discarded the flow through, and reused the column for the next step. Secondly, we pipetted a 500 μl buffer RPE to the spin column and again centrifuged it at 8,000 ×*g* at room temperature for 15 s, discarded the flow through water, and repeated the previous step. To finish, we transported the spin column to the new collecting tube and centrifuged the tube for 2 min at full speed for dehydration, and for elusion, the column was transferred to a clean Eppendorf tube and 50 ul Rnase-free water was pipetted directly onto the column and centrifuged for 1 min at 8,000 ×*g*. A NanoDrop (ND)-1000 spectrophotometer (NanoDrop Technologies, Inc. Wilmington, DE, USA) was used to quantify extracted RNA purity and concentration; about 1–2 μl of eluted RNA samples were sufficient, and the ratio of absorbance at 260 and 280 nm is used to assess the purity of RNA; a ratio of ∼2.0 is generally accepted as “pure” for RNA.

Reverse transcription to obtain cDNA was performed using the RT2 First Strand Kit (Qiagen, Valencia, CA, USA) according to the manufacturer’s protocol in a final volume of 20 μl (10 μl reverse-transcription mix was added to each tube containing a genomic DNA elimination mix that has the extracted RNA and DNA elimination buffer).

The expression of lncRNAs, including target genes, in serum, has been previously documented (Shaker et al., 2019a; Shaker et al., 2019b). The expression of lncRNAs *NEAT1* and *lnc-DC* in serum was measured using the RT2 SYBR Green PCR kit (Qiagen, Germantown, MD, USA) using predesigned primers obtained for *NEAT1* (Qiagen, Valencia, CA, USA, Catalog no: 330701 LPH15809A, Accession no: NR_028272.1), *lnc-DC* (Catalog no: 330701 LPH23184A, Accession no: NR_030732.1), and *GAPDH* as an internal housekeeping gene (Adriaens et al., 2016) (Catalog no: 330701 LPH31725A, Accession no: ENST00000496049.0).

Quantitative real-time PCR was carried out by the Rotor-gene Q real-time PCR system (Qiagen, USA) under the following conditions: 95°C for 10 min, followed by 45 cycles at 95°C for 15 s and 60°C for 60 s. The relative fold changes of the target genes in patients affected by BD compared to the controls were calculated using the 2^−ΔΔCt^ equation (Livak and Schmittgen 2001). For the control sample, ΔΔCt equals zero, and 2^0^ equals one (Shaker et al., 2019b).

### Sample Size

The sample size was calculated using G power version 3.0.10. The minimum sample size of patients needed to get a power level of 0.80, an alpha level of 0.05, and a medium effect size of 0.50 for *NEAT1* and *lnc-DC* was 52 in each group.

### Statistical Analysis

The collected data were organized, tabulated, and statistically analyzed using SPSS software package version 22 (SPSS Inc., Chicago, IL, USA). Age and other basic data were presented as mean and standard deviation (SD). Independent *t*-test was used to test significance. *NEAT1* and *lnc-DC* data were presented as median and interquartile range (IQR). The Mann–Whitney U and Kruskal–Wallis tests were used to compare between any two or three groups, respectively. Spearman correlation was performed to identify any relationships between *NEAT1* and *lnc-DC* with study parameters among patient cases. ROC curve analysis was used to determine the cut-off point differentiating between case and control with the highest sensitivity and specificity for *NEAT1* and *lnc-DC*. Categorical data were presented as frequencies and percentages, and chi-square (*χ* ^2^) was used as a test of significance. For the interpretation of results, the significance was determined to be *p* ≤ 0.05.

## Results

### Basic Characteristics of BD Patients and Controls

The demographic data of the studied groups are presented in Table 1. This case-control study was conducted on 52 (76.9% males, 23.1% females) BD patients aged 38.1 ± 2.3 and with a disease duration of 7.2 ± 6.6 years compared with 52 healthy controls with matched age (39.2 ± 3.3, *p* = 0.524) and sex (75% males, 25% females, *p* = 0.819).

**TABLE 1:** Basic characteristics of BD and control groups.

Variables	BD (*n* = 52)	Healthy controls (*n* = 52)	*p*-value
	**Mean ± SD**		
Age, years	38.1 ± 2.3	39.2 ± 3.3	0.524^#^
Sex, female *n* (%)/male *n* (%)	12 (23.1%)/40 (76.9%)	13 (25.0%)/39 (75.0%)	0.819^##^
Duration, years	7.2 ± 6.6	—	—
Pulse rate/min	72.4 ± 6.8	—	—

BD, Behçet’s disease; ^#^Independent-*t* test; ^##^Chi-squared test.

### Clinical Characteristics of Patients

Family history, clinical data, activity index, and treatment options are presented in Table 2. Out of 52 BD patients, eight (15.4%) had a positive family history, 16 (30.8%) had a positive pathergy test, and 36 (69.3%) had an active disease, with a score of 1 (38.5%), 2 (23.1%), or 3 (7.7%) on the activity index. All patients suffered from oral ulcers, 92.3% from genital ulcers, 38.5% from cutaneous lesions (erythema nodosum), and 61.5% from joint manifestations (polyarthralgia). Ocular examination revealed that 53.8% of patients had uveitis. Vascular and CNS assessments showed that 23.1% of patients had vascular manifestations (deep venous thrombosis, pulmonary embolism, or subclavian aneurysm), while 38.5% had neurological manifestations (migraine headache, hemiplegia, or cerebral infarctions).

**TABLE 2:** Clinical characteristics of patients.

**Parameter**		***n* (%)**
Family history	Positive	8 (15.4%)
Oral ulcer	Present	52 (100%)
Genital ulcer	Present	48 (92.3%)
Cutaneous lesions	Erythema nodosum	20 (38.5%)
Ocular lesions	Uveitis	28 (53.8%)
Vascular lesions	(DVT, pulmonary embolism, subclavian aneurysm)	12 (23.1%)
Articular lesions	Polyarthralgia	32 (61.5%)
Neurological lesions	(Migraine headache, cerebral infarction, hemiplegia)	20 (38.5%)
Pathergy test	Positive	16 (30.8%)
Activity	Yes	36 (69.2%)
BDCAI	0	16 (30.7%)
	1	20 (38.5%)
	2	12 (23.1%)
	3	4 (7.7%)
Steroids	Yes	52 (100%)
Azathioprine	Yes	48 (92.3%)
Colichicine	Yes	52 (100%)

BDCAI, Behçet’s disease current activity index; DVT, deep vein thrombosis. *p* values < 0.05 are statistically significant.

### Expression of *NEAT1* and *lnc-DC* in the BD Patient Group Relative to Healthy Controls

**FIGURE 1:**
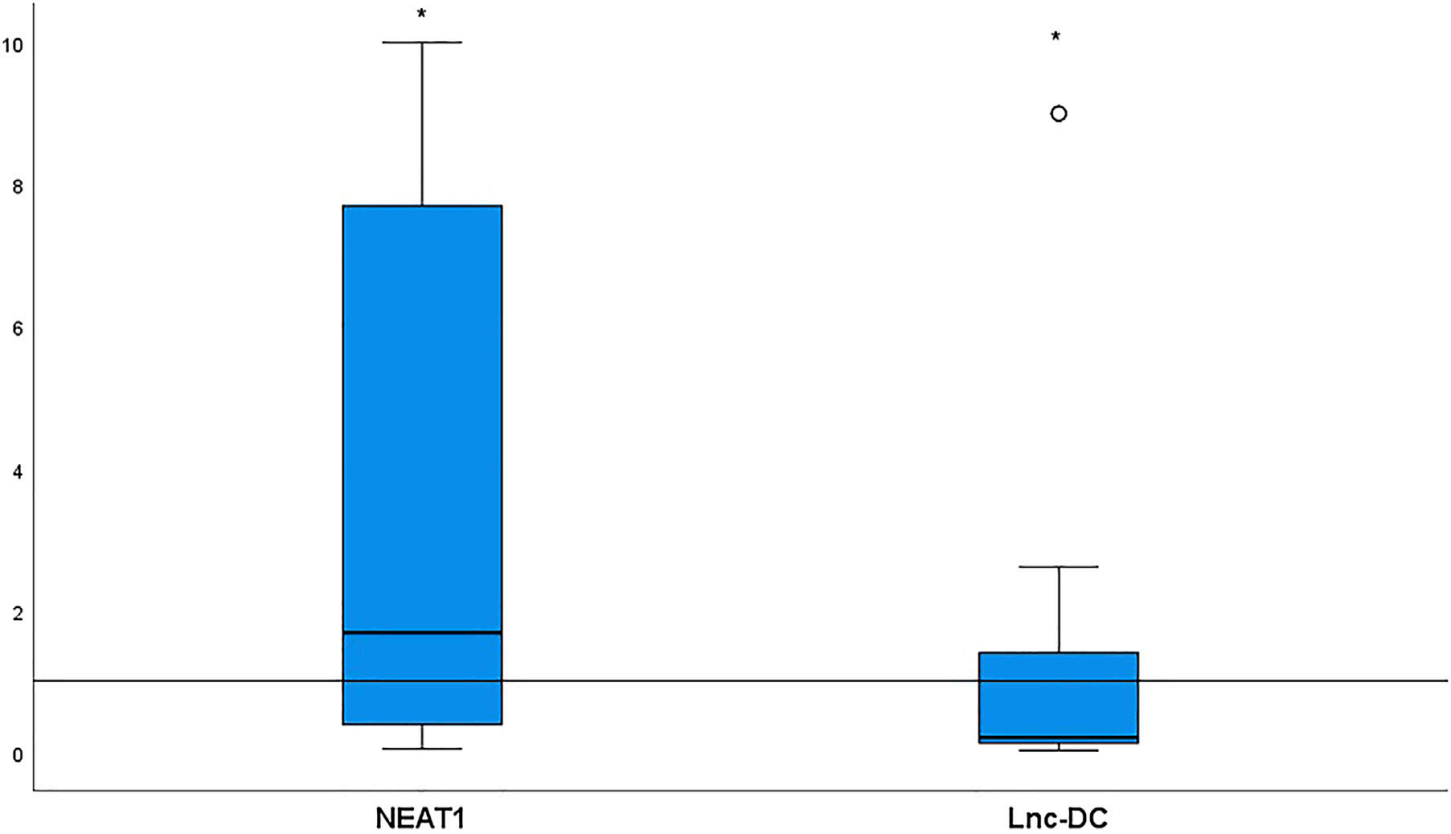
NEAT1 and lnc-DC expressions in serum from 52 BD patients relative to healthy controls. Data are represented by box plot (median, upper, and lower quartiles). The horizontal dotted line represents transcript expression in the control group, which is 1 according to the 2^−ΔΔCt^ equation. The increase of *NEAT1* (*p* < 0.0001) and the decrease of *lnc-DC* (*p* = 0.03) were detected in total BD patients vs. healthy controls. *Significant.

Relative target gene expression is shown in Figure 1. Significantly more *NEAT1* [median (IQR) = 1.68 (0.38–7.7), *p <* 0.0001] and significantly less *lnc-DC* [median (IQR) = 0.2 (0.12–1.39), *p* = 0.03] were detected in the sera collected from BD patients relative to the controls.

### Relationship Between *NEAT1*, *lnc-DC*, and Patient Demographics and Clinical Data

The relationships between *NEAT1*, *lnc-DC*, and patient demographics and clinical data were analyzed. We determined that higher serum expression levels of *NEAT1* and *lnc-DC* were significantly associated with the following clinical presentations: cutaneous lesions, vascular manifestations, articular manifestations, neurological manifestations, and higher activity score. *NEAT1* was also significantly associated with a negative pathergy test, and higher *lnc-DC* was significantly associated with a positive family history (Table 3).

**TABLE 3:** Gene expression in relation to demographic and clinical characteristics among cases.

Parameter		*NEAT1*	*p*-value	*lnc-DC*	*p*-value
		Median (IQR)		Median (IQR)	
Sex	Female	1.92 (1.27–4.74)	0.486^a^	0.33 (0.12–2.6)	0.296^a^
	Male	1.43 (0.11–8.84)		0.19 (0.04–1.39)	
Family history	Negative	1.68 (0.11–7.7)	0.233^a^	0.17 (0.04–1.13)	0.003^a^*
	Positive	5.33 (1.18–9.48)		1.5 (1.39–1.6)	
Genital ulcer	Absent	1.68 (1.5–2.35)	1.000^a^	0.18 (0.17–0.2)	0.607^a^
	Present	1.6 (0.25–8.27)		0.26 (0.08–1.5)	
Cutaneous lesions	Absent	0.83 (0.07–4.69)	<0.0001^a^*	0.13 (0.04–0.19)	<0.0001^a^*
	Present	4.74 (1.92–9.48)		1.6 (1.39–2.6)	
Ocular lesions	Absent	1.6 (1.18–4.74)	0.377^a^	0.73 (0.12–1.39)	0.376^a^
	Present	1.68 (0.11–9.48)		0.17 (0.04–1.6)	
Vascular lesions	Absent	1.23 (0.11–7.7)	0.015^b^*	0.15 (0.04–1.39)	0.037^b^*
	Present	4.74 (1.92–8.84)		0.33 (0.20–2.6)	
Articular lesions	Absent	0.38 (0.11–1.27)	0.007^a^*	0.12 (0.04–0.13)	<0.0001^a^*
	Present	3.33 (1.43–8.59)		1.26 (0.25–2.1)	
Neurological lesions	Absent	0.78 (0.07–4.48)	<0.0001^b^*	0.13 (0.04–1.26)	0.001^b^*
	Present	4.74 (1.92–8.84)		0.33 (0.20–2.6)	
Pathergy test	Negative	1.92 (1.27–7.7)	0.011^a^*	0.2 (0.12–1.13)	1.000^a^
	Positive	0.61 (0.04–5.33)		0.76 (0.08–1.5)	
Activity	No	3 (0.69–6.79)	0.750^a^	0.16 (0.07–1.4)	0.203^a^
	Yes	1.68 (0.38–7.7)		0.33 (0.13–1.39)	
BDCAI	0	3 (0.69–6.79)	<0.0001^b^*	0.16 (0.07–1.4)	<0.0001^b^*
	1	0.38 (0.04–1.18)	0 vs. 1: 0.039	0.13 (0.04–0.17)	0 vs. 3: 0.006
	2	7.7 (1.92–9.48)	0 vs. 3: 0.039	1.13 (0.33–1.6)	1 vs. 2: 0.016
	3	9.79 (5.36–10.73)	1 vs. 2: <0.0001 1 vs. 3: <0.0001	9.05 (8.86–9.18)	1 vs. 3: 0.001
Azathioprine	No	1.18 (1.09–1.21)	0.292^a^	1.39 (1.01–2.07)	0.105^a^
	Yes	1.8 (0.25–8.27)		0.19 (0.08–1.37)	

BDCAI, Behçet’s disease Current Activity Index; IQR, interquartile range; *lnc-DC*, lncRNA in dendritic cells; *NEAT1*, nuclear-enriched abundant transcript 1.

a Mann–Whitney U test.

b Kruskal–Wallis test.

*Significant. *p* values < 0.05 are statistically significant.

### Correlation Analysis Between *NEAT1* and *lnc-DC* and Patient Numerical Data

There was a strong positive correlation between the two studied genes (*r* = 0.802, *p* < 0.0001). *NEAT1* and *lnc-DC* expressions also showed significant positive correlation with disease duration (*r* = 0.318, *p =* 0.022 for *NEAT1*, *r* = 0.299, *p =* 0.031 for *lnc-DC*), pulse rate (*r* = 0.347, *p =* 0.012 for *NEAT1*, *r* = 0.391, *p =* 0.004 for *lnc-DC*), and with BDCAI activity score (*r* = 0.390, *p =* 0.004 for *NEAT1*, *r* = 0.473, *p <* 0.0001 for *lnc-DC*). However, the patient’s age was only significantly positively correlated with *NEAT1* (*r* = 0.402, *p =* 0.003) (Table 4).

**TABLE 4:** Correlation analysis between *NEAT1* and *lnc-DC* and numerical data of patients.

**Variable**	***NEAT1***		*l**nc-DC***	
	***r***	***p*-value**	***R***	***p*-value**
*lnc-DC*	0.802	<0.0001*		
Age/years	0.402	0.003*	0.259	0.064
Duration, years	0.318	0.022*	0.299	0.031*
Pulse rate/min	0.347	0.012*	0.391	0.004*
BDCAI	0.390	0.004*	0.473	<0.0001*

BDCAI, Behçet’s disease Current Activity Index; *lnc-DC*, lncRNA in dendritic cells. *Significant. *p* values < 0.05 are statistically significant.

### ROC Analysis to Determine the Diagnostic Performance of *NEAT1* and *lnc-DC*

**FIGURE 2:**
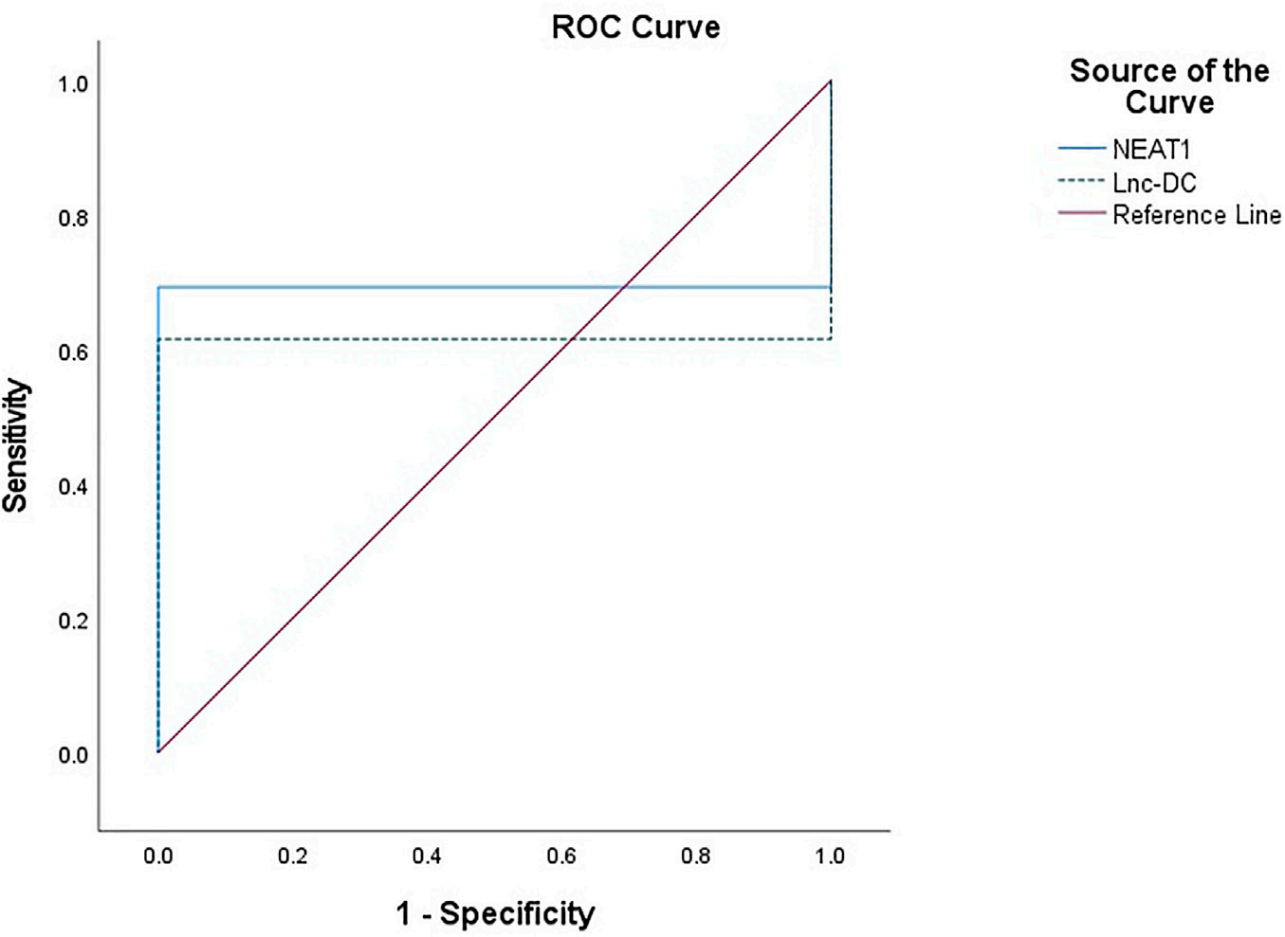
ROC curve to predict the diagnostic performance of *NEAT1* and *lnc-DC* found in serum for differentiating BD patients from controls. ROC curves showed that the expression of *NEAT1* and *lnc-DC* in serum could be used as predictors of BD. *NEAT1* had an area under the curve (AUC) of 0.692 (95% CI: 0.591–0.794, *p* = 0.001), and *lnc-DC* had an AUC of 0.615 (95% CI: 0.508–0.723, *p* = 0.043).

The expression of *NEAT1* and *lnc-DC* in serum had diagnostic value. RNA levels of these two genes could be used to distinguish BD patients from healthy controls. For *NEAT1*, AUC = 0.692, 95% confidence interval (CI) = 0.591–0.794, cut-off point = 1.09, *p* = 0.001, with sensitivity = 69.2%, and specificity = 100%. For *lnc-DC*, AUC = 0.615, 95% CI = 0.508–0.723, cut-off point = 0.66, *p* = 0.043, with sensitivity = 61.5%, and specificity = 100% (Table 5; Figure 2).

**TABLE 5:** ROC curve analysis to determine the diagnostic performance of *NEAT1* and *lnc-DC.*

**Variable**	**AUC (95% CI)**	***p*-value**	**Cut-off point**	**Sensitivity% (%)**	**Specificity% (%)**
*NEAT1*	0.692 (0.591–0.794)	0.001*	1.09	69.2	100.0
*lnc-DC*	0.615 (0.508–0.723)	0043*	0.66	61.5	100.0

AUC, area under the curve; *lnc-DC*, lncRNA in dendritic cells; *NEAT1*, nuclear-enriched abundant transcript 1. *Significant. *p* values < 0.05 are statistically significant.

## Discussion

BD is a rare systemic autoimmune vasculitis disease with self-limiting recurrent inflammatory attacks. The recurrent attacks involve vital organs, leading to secondary serious consequences such as uveitis, optic atrophy and blindness, cerebral venous thrombosis, aseptic meningitis, hemiplegia, deep venous thrombosis, pulmonary embolism, and major artery aneurysms. An early diagnosis of BD is very important to avoid fatal complications, and as there is no specific diagnostic test for BD, there is a pressing need to identify sensitive biomarkers to aid in rapid diagnosis and serve in monitoring disease activity and response to treatment (Zeidan et al., 2016; Leccese and Alpsoy 2019).

Recently, increasing evidence indicates that the lncRNAs found in blood serum are dysregulated in numerous autoimmune diseases and could be used as the identifying the biomarkers and novel therapeutic targets of those diseases (Santoro et al., 2016; Shaker et al., 2019b). Up to now, little was known about the importance of lncRNAs in BD, and no studies had been performed to uncover any role(s) that may be played by *NEAT1* and/or *lnc-DC* in BD.

In the present study, we showed that the *NEAT1* expression level was increased significantly in the serum from patients with BD relative to controls.

Previous studies have demonstrated a robust relationship between *NEAT1* and the inflammatory process. The upregulation of *NEAT1* has been shown to promote the release of several inflammasomes (NLRP3, NLRC4, and AIM2) and pro-inflammatory cytokines (CXCL10, IL-6, and IL-1β), resulting in an immune response in inflammatory and immune diseases (Imamura et al., 2014; Zhang et al., 2016; Huang et al., 2017; Pandey et al., 2017; Huang et al., 2018; Prinz et al., 2019; Zhang et al., 2019). For example, the upregulation of *NEAT1* was recently reported in bronchial asthma (Li, Ye, and Lu 2020) and SLE (Zhang et al., 2016). In addition, several studies have shown that *NEAT1* overexpression results in elevated levels of reactive oxygen species, which worsen immune reactions and exacerbate inflammatory reactions (Wang et al., 2019). Because of its role(s) in immune response, it has been suggested that *NEAT1* could be used as a therapeutic target for inflammatory and autoimmune diseases (Imamura et al., 2014; Zhang et al., 2016; Huang et al., 2017; Pandey et al., 2017; Prinz et al., 2019; Zhang et al., 2019).

Considering the previous evidence, we hypothesized that *NEAT1* may be implicated in the immune and inflammatory responses occurring in BD.

Our results showed that the expression of *NEAT1* is markedly elevated in patients who have skin lesions, neurological symptoms, vascular involvement, and articular involvement (arthritis). Moreover, it is interesting that *NEAT1* positively correlated with disease activity score (BDCAI), demonstrating that increased inflammation may lead to higher expression of *NEAT1*.

Our findings agree with Zhang et al. (2016) who found a positive correlation between the *NEAT1* expression and disease activity in SLE. In addition, Wang et al. (2017) reported that the inhibition of *NEAT1* results in the improvement of skin lesions associated with herpes simplex infection. *NEAT1* is also hypothesized to contribute to neurodegenerative diseases (Michelhaugh et al., 2011; Nishimoto et al., 2013; Zhong et al., 2017). Interestingly, *NEAT1* is also abundant in the rat model of osteoarthritis (OA) and contributes to the disease *via* different mechanisms that result in the increased production of inflammatory cytokines and the increased apoptosis of chondrocytes (Wang et al., 2017; Zhang et al., 2020a).

In the present study, our results showed that the *lnc-DC* expression in serum is markedly reduced in BD patients relative to controls. However, higher levels of *lnc-DC* are significantly associated with the presence of cutaneous, vascular, articular, and neurological lesions; the presence of family history; and a higher activity score (BDCAI). The serum level of *lnc-DC* was also positively correlated with the duration of disease and pulse rate.

*lnc-DC* is exclusively expressed in human conventional DCs, promoting their differentiation and increasing the capacity of DCs to stimulate T cell activation. *lnc-DC* mediates these effects by activating the transcription factor STAT3 (Wang et al., 2014). STAT3 regulates IL-21 expression, and STAT3-deficient T helper (Th) cells fail to produce IL-21 mRNA and protein. Previous literature suggests that IL-21 may contribute to the pathogenesis of BD by disrupting the equilibrium between Th17 cells and regulatory T cells (Tregs). triggering an autoimmune response in BD patients (Zeidan et al., 2016). This is because Th17 cells cause autoimmunity and inflammation, whereas Treg cells inhibit these phenomena and maintain immune homeostasis (Lee 2018). In turn, we propose that *lnc-DC* is implicated in the pathogenesis of BD because increased *lnc-DC* was related to more active diseases and the presence of their manifestations.

The reduced *lnc-DC* in serum from patients in our study relative to the controls could be explained by the reduced number of DC cells present in the blood of patients with autoimmune diseases compared with that of healthy controls, while concurrently, the number of DCs in target tissues of individuals with autoimmune disease increases (Coutant and Miossec 2016). The aforementioned findings suggest that circulating DCs and, in turn, *lnc-DC* are reduced in the blood obtained from patients with autoimmune diseases. Another explanation for this finding is that the lncRNAs in serum could be fragmented because of their very long sequence lengths and so are unlikely to exist in a full-length form in the serum (Ren et al., 2013; Wang et al., 2015).

In recent publications’ results, *lnc-DC* levels were shown to be markedly downregulated in patients with RA and SLE (Li et al., 2017; Zhang et al., 2019) which is consistent with our results concerning BD patients. Meanwhile, Shaker et al. (2019) suggest that *lnc-DC* can be used as a biomarker for the diagnosis of multiple sclerosis (MS) since they observed a high level of *lnc-DC* in the serum of patients with MS. The discrepancy between upregulation vs. downregulation of *lnc-DC* in different autoimmune diseases is hard to definitively clarify as few studies examine the role(s) of *lnc-DC* in the pathogenesis of autoimmune diseases. The differences in lncRNA expression may be due to tissue specificity in the expression of some lncRNAs (Cabili et al., 2011).

Higher *lnc-DC* expression was associated with positive family history. Possible explanations for this relationship include that the familial accumulation of BD has been documented in 1–18% of the population with a higher incidence of familial association in juvenile patients (Zeidan et al., 2016) or that the loci transcribing novel lncRNAs co-localize with leukocyte transcriptional enhancers near genetic variants associated with autoimmune disease risk, Moreover, the modifications to enhance function, including lncRNA expression, produced by genetics and environmental stimulants can change cellular phenotypes contributing to disease susceptibility and pathogenesis (Aune et al., 2017).

A positive correlation between *NEAT1* and *lnc-DC* enforces their synergistic effects. ROC curve analysis was performed to explore the diagnostic value of lncRNAs *NEAT1* and *lnc-DC* as predictors for discrimination between BD patients and controls. At cut-off points of 1.07 and 0.66 for *NEAT1* and *lnc-DC*, respectively, both biomarkers had an excellent specificity (100% for both) and fair sensitivity (69.2 and 61.5% for *NEAT1* and *lnc-DC*, respectively). The AUC for *NEAT1* and *lnc-DC* was 0.692 and 0.615, respectively.

The results presented here offer a potential new significance for lncRNA *NEAT1* and *lnc-DC* in the prediction of BD. Future studies are required to verify our results and elucidate the involvement of *NEAT1* and *lnc-DC* in the pathogenesis of BD. Such future studies are needed to overcome the limitations of the current study, which include 1) small sample size due to the rare pattern of the disease and 2) the lack of scientific literature illustrating the exact role or underlying molecular contributions of lncRNA generally and of *NEAT1* and *lnc-DC* specifically in the pathogenesis, propagation, and prognosis of BD. Larger-scaled studies that explore the exact functions of these genes in BD pathogenesis are required.

## Conclusion

We showed that *NEAT1* and *lnc-DC* can be used as novel biomarkers for BD. In addition, their high transcript levels were associated with disease activity score and could be targets of therapy in patients with BD.

## Data Availability

The original contributions presented in the study are included in the article/Supplementary Material, further inquiries can be directed to the corresponding author.
