# Supplementary material for: Expression of lncRNAs NEAT1 and lnc-DC in Serum From Patients With Behçet’s Disease Can Be Used as Predictors of Disease
Source: Front Mol Biosci. 2022 Jan 19;8:797689. doi: 10.3389/fmolb.2021.797689 (PMC8809491; doi:10.3389/fmolb.2021.797689)
Supplement: Supplementary file 1 [file DataSheet1.docx]

**B) Laboratory methods for detection of NEAT1 and lnc-DC in serum:**

1. **RNA extraction:**

This step was performed using miRNeasy mini kit and protocol for purification of serum total RNA, including long noncoding RNA (Qiagen, Valencia, CA, USA).

**Principles:**

The miRNeasy Mini Kit combines phenol/guanidine-based lysis of samples and silica membrane-based purification of total RNA. QIAzol Lysis Reagent is designed to facilitate lysis of samples, to inhibit RNases, and to remove most of the cellular DNA and proteins from samples by organic extraction.

Addition of chloroform separates the homogenate into upper aqueous layer in which the RNA concentrates, an intermediate layer contains DNA, and third lower organic layer of denatured proteins.

The upper, aqueous layer is extracted, and ethanol is added to provide appropriate binding conditions for all RNA molecules. The sample is then applied to the RNeasy Mini spin column, where the total RNA binds to the membrane and phenol and other contaminants are efficiently washed away. RNA is then eluted in RNase-free water.

**Reagents**

- QIAzol Lysis Reagent (50mL).
- Washing buffer RWT concentrate (18mL).
- Washing buffer RPE concentrate (11mL).
- RNase-free water (10mL).

**Procedure:**

1. One mL QIAzol lysis reagent was added to 200 μL serum and incubated for 5 min at room temperature.

2. Two hundred μL chloroform was added.

3. The mixture was shaken vigorously using vortex for 15 seconds.

4. Incubation was done for 2-3 min at room temperature.

5. Centrifugation at 12000 xg at 4°C was done for 15 min.

6. The upper watery phase was removed and 1.5 times of its volume 100% ethanol was added.

7. Seven hundred uL of this mixture was placed in RNeasy Mini spin column in 2 ml collection tube and centrifuged at 8000 xg at room temperature for 15 sec.

8. After the mixture had completely passed the column, 700 μL of buffer RWT was added to each column, and again centrifuged at 8000 xg at room temperature for 15 sec.

9. Five hundred μL buffer RPE was added to the column and centrifuged at 8000 xg at room temperature for 15 sec.

10. The previous process was repeated.

11- centrifugation for 2 min at full speed (14,000xg).

11. The column was transferred to new 1.5 ml collection tube and 50 uL RNase-free water was pipetted directly onto the column and centrifuged for 1 min. at 8000 xg to elute RNA.

**B) Quantitation and assessment of RNA purity:**

RNA samples were subjected to RNA quantitation and purity assessment using the NanoDrop® (ND)-1000 spectrophotometer (NanoDrop Technologies, Inc. Wilmington, USA).

I) Blanking and Absorbance Calculations

1. The NanoDrop ND-1000 Spectrophotometer is ‘‘blanked’’ with 1 μL RNase free water, a spectrum is taken from it as a reference material (blank) and stored in memory as an array of light intensities by wavelength.
2. Measurement of a sample is taken where the intensity of light that has transmitted through the sample is recorded.
3. The sample absorbance was calculated according to the following equation:

Absorbance= -log (Intensity _sample_/Intensity _blank_)

The readings ratio at 260 nm and 280 nm offers an estimate of RNA purity. RNA is considered pure when the A260/A280 ratio is 1.8–2.1.

**C) Reverse transcription (RT) of RNA into complementary DNAs (cDNAs):**

Reverse transcription was carried out on total RNA in a ﬁnal volume of 20 uL RT reactions using the RT2 First Strand Kit (Qiagen, Valencia, CA, USA) as follows:

1. The reagents of the RT2 First Strand Kit (10–15 seconds) were briefly centrifuged to bring the contents to the bottom of the tubes.

2. The genomic DNA elimination mix was prepared for each RNA sample in a sterile PCR tube according to the following Table . Gently mixing was done with centrifugation.

| **Table 1. Genomic DNA elimination mix Component** | **Amount** |
| --- | --- |
| RNA | 25 ng – 5 μg |
| Buffer GE | 2 μl |
| Nuclease-Free Water | Variable |
| **Total volume** | **10 μl** |

**3**. The genomic DNA elimination mix was Incubated for 5 minutes at 42°C, then place immediately on ice for at least 1 minute.

1. **The reverse-transcription master mix was prepared on ice according to Table shown below.**

| **Table 2. Reverse-transcription mix Component** | **Volume for 1 reaction** |  |  |
| --- | --- | --- | --- |
| 5x Buffer BC3 | 4 μl |  |  |
| Control P2 | 1 μl |  |  |
| RE3 Reverse Transcriptase Mix | 2 μl |  |  |
| Nuclease-Free Water | 3 μl |  |  |
| **Total volume** | **10 μl** |  |  |

5. 10 μl reverse-transcription mix was added to each tube containing genomic DNA elimination mix. Gently mixing was done by pipetting up and down.

6. Incubation was done for 60 min at 37ºC using conventional PCR.

7. Incubation for 5 min at 95ºC was done to inactivate Reverse Transcriptase using conventional PCR.

**F) Quantitative Real-time PCR (qPCR) for Detection of long non coding RNA NEAT1 and lnc-DC:**

1- The reagents of RT2 SYBR Green PCR Mastermix, RT2 IncRNA qPCR Assay (Qiagen, Maryland, USA), and cDNA synthesis reaction were briefly centrifuged (10–15 seconds) to bring the contents to the bottom of the tubes.

2. Reaction mix was prepared in a nuclease-free tube according to Table below for a 25μl per well reaction volume.

| **Table 4. PCR components per reaction Component** | **Volume for 1 reaction** |  |
| --- | --- | --- |
| Nuclease-Free Water | 10.5 μl |  |
| RT2 SYBR Green Mastermix | 12.5 μl |  |
| cDNA | 1 μl |  |
| RT2 IncRNA qPCR Assay (10 μM stock) | 1 μl |  |
| **Total volume** | **25 μl** |  |

3. The real-time cycler Rotor-gen Rotor-gene Q Real-time PCR system (Qiagen, USA) e Q Real-time PCR system (Qiagen, USA) was programmed according to table below.

The expression of lncRNAs *NEAT1* and *lnc-DC* in serum was measured using predesigned primers obtained for *NEAT1* (Qiagen, Valencia, CA, USA, Catalog no: 330701 LPH15809A, Accession no: NR_028272.1), *lnc-DC* (Catalog no: 330701 LPH23184A, Accession no: NR_030732.1), and *GAPDH* as an internal housekeeping gene (Catalog no: 330701 LPH31725A, Accession no: ENST00000496049.0).

The cycle threshold (Ct) is the number of cycles required for the fluorescent signal to cross the threshold in real-time PCR. Gene expression relative to internal control (2−ΔCt) was calculated. Fold change was calculated using 2−ΔΔCt.

- - 1. **Calculation of results:**

After completion of the PCR cycles, melting curve analyses were performed to validate the specific generation of the expected PCR product. GAPDH was used to normalize the expression pattern and for relative quantification of the target Long non coding RNA.

The cycle threshold (*Ct*) value is the number of qPCR cycles required for the fluorescent signal to cross a specified threshold.

Δ*Ct* was calculated by subtracting the *Ct* values of GAPDH from those of target long non coding RNAs (*NEAT1* and *lnc-DC*).

ΔΔ*Ct* was calculated by subtracting the Δ*Ct* of the control samples from the Δ*Ct* of the disease samples.

The fold change in *NEAT1* and *lnc-DC* expressions were calculated by the equation 2^–ΔΔ^*^Ct^*.

The mathematical relationship between *Ct*, Δ*Ct,* ΔΔ*Ct* and FC (Rq) is:

Δ*Ct_(patients)_ = Ct _(long non coding RNA)_ – Ct _(Endogenous control)_*

Δ*Ct_(control)_ = Ct _(long non coding RNA)_ – Ct _(Endogenous control)_*

ΔΔ*Ct_(patients)_ =* Δ*Ct_(patients)_ –* Δ*Ct_(Control)_*

*FC (Rq) = 2^-^*^ΔΔ^*^Ct^*

If the FC is more than 1 it means that the long non coding RNA is upregulated; if the FC is less than 1 it means it is downregulated. Control value was assumed equaled 1, because –ΔΔ*Ct* for control subjects equals zero and 2^0^ equals one.
